# Curious Action of Colchicum on the Urine

**Published:** 1829-01-01

**Authors:** 


### ACTION OF COLCHICUM ON THE URINE.

Professor Chelius, of Heidelberg, has been making a number of experiments on the urine of those who take colchicum for different complaints, and has discovered a very curious and uniform effect to result from this powerful medicine—namely, a great increase of uric acid in the urine—and, consequently, a great evacuation of it from the system at large. Thus a man, who had rheumatic inflammation and swelling of several joints, was put upon the use of colchicum, his urine being first examined, and found to contain 0,069 of uric acid, free or combined with ammonin. In four days after the colchicum was commenced, and that in moderate doses, it rose from 69 to 76. On the eighth day it was 91, and on the 12th day it was 102. In short, in the course of a fortnight, the quantity of uric acid was doubled. Experiments were made on a great number of individuals, chiefly labouring under gouty, rheumatic, and neuralgic affections, and all with the same result—a great increase of the uric acid. Professor Chelius thinks, that this operation of the colchicum may lead to some elucidation of the specific efficacy of the medicine in various diseases, and perhaps may throw some light on the nature of those diseases themselves. The Professor appears to employ the colchicum, with great success, in neuralgiæ of the face, in sciatica, rheumatic ophthalmia, dropsy of the joints, and in certain paralytic affections of the lower extremities connected with the arthritic diathesis. We would recommend the medical practitioners of this country to note the effects of colchicum on the urine; as the increase of uric acid may lead to the useful employment of this remedy in some diseases to which it is not now applied.

